# Editorial: Zoonotic diseases among pigs

**DOI:** 10.3389/fvets.2022.1122679

**Published:** 2023-01-05

**Authors:** Chao-Nan Lin, Tamaki Okabayashi, Padet Tummaruk, Peck Toung Ooi

**Affiliations:** ^1^Department of Veterinary Medicine, College of Veterinary Medicine, National Pingtung University of Science and Technology, Pingtung, Taiwan; ^2^Center for Animal Disease Control, University of Miyazaki, Miyazaki, Japan; ^3^Faculty of Veterinary Science, Chulalongkorn University, Bangkok, Thailand; ^4^Faculty of Veterinary Medicine, University Putra Malaysia, Serdang, Malaysia

**Keywords:** veterinary infectious diseases, zoonoses, emerging diseases, pigs, virus, bacteria

The pig is a domestic animal that is closely related to humans. It provides essential meat protein to our diet, acts as a companion animal, and serves as an essential animal model for studies and research. Since husbandry modernization, the pig rearing system developed into large-scale and intensive farming to adequately supply the needs of a growing human population, thus increasing the risk of zoonosis. A zoonotic disease is an infectious disease that is transmitted between animals and humans. Unfortunately, most emerging infectious diseases detected in pigs over the last four decades have been zoonotic-related. Most zoonoses are often previously unidentified diseases, such as coronavirus disease 2019 (COVID-19), swine-origin H1N1 (2009), and Nipah virus (1999).

It is known that almost two-thirds of the pathogens that cause diseases in humans are of animal origin. Among zoonoses, some diseases associated with pigs have been reported, such as Japanese encephalitis virus (JEV), hepatitis E virus (HEV), Nipah virus (NiV), swine influenza virus (SIV), *Clostridium difficile, Streptococcus suis, Leptospira* spp., *Salmonella* spp., *Brucella* spp., *Trichinella spiralis*, and others. Of these, NiV and SIV are emerging diseases. Additionally, the pig is known as the amplifier host for JEV, NiV, and others. The pathogens are enhanced once they infect pigs, which increases their zoonotic potential. Several NiV outbreaks appeared after human contact with excreta, such as saliva, urine, and feces of sick pigs or bats. The human-to-human transmission of these diseases related to pigs has also been reported. The treatment of human patients with zoonoses infection remains dependent on supportive care. Most transmission routes of zoonoses in humans are associated with direct exposure to infected pigs, or infected raw or undercooked pork products. In addition, antimicrobial resistance has become a global threat to public health in the last two decades, including antimicrobial resistance of zoonotic spp. Therefore, there is a need to focus on zoonotic diseases among livestock, including in pigs.

The articles included in this Research Topic focused on factors that impact the health between pigs and humans. The aim of the Research Topic was to describe the most recently discovered pathogenesis, molecular evolution, immune responses, transmission, treatment, control, and prevention of zoonotic pathogens in pigs. Five manuscripts were published under this Research Topic.

The first manuscript, titled “*Effect Of Porcine Clostridium perfringens on intestinal barrier, immunity, and quantitative analysis of intestinal bacterial communities in mice*” (Jiang et al.), was written by corresponding authors Zeqing Lua and Yizhen Wang, from the research group of Zhejiang University, Hangzhou. The research group focused on *Clostridium perfringens*, a gram-positive, rod-shaped bacterium that can be isolated from a broad range of environments, including the gastrointestinal tract of humans and animals. It was reported that *C. perfringens* bacteria are one of the most common causes of foodborne illness (food poisoning) (1). The Centers for Disease Control and Prevention (CDC) estimated these bacteria cause ~1 million illnesses in the United States every year (2). This research work evaluated the effects of different porcine *C. perfringens* dose (High: 2.0 × 109 CFU/mL, Medium: 4.0 × 108 CFU/mL, Low: 8.0 × 107 CFU/mL) treatments in mice by oral gavage. Its results also provided for an experimental and theoretical basis for the construction of porcine *C. perfringens* infection model in mice (Jiang et al.).

The next manuscript was “*Environment and offspring surveillance in porcine brucellosis*” (Rebollada-Merino et al.), written by corresponding author Marta Pérez-Sancho from the research group of the Complutense University of Madrid, Madrid, Spain. The research group's study on *Porcine brucellosis*, as the pathogenesis and epidemiology of brucellosis in swine, were not widely characterized, and the zoonotic potential remains high in the community (3). The research work was the first study describing environmental surveillance and *Brucellosis suis* DNA presence at porcine farm facilities (Rebollada-Merino et al.). The research team suggested that the environmental approach was a more simple, practical, valuable, and safe method to detect and monitor *B. suis*, and, thus, should be considered in porcine brucellosis surveillance and control programs (Rebollada-Merino et al.).

The third manuscript was “*Propidium monoazide combined with RT-qPCR detects infectivity of porcine epidemic diarrhea virus*” (Liang et al.) by corresponding authors Gong Liang, Chao Huang, and Xibiao Tang, from the research team of the Diagnostic Center Department, Wuhan Keqian Biology Co., Ltd., Wuhan, China. Currently, there is no established test method to detect the infection of the porcine epidemic diarrhea virus (PEDV). In the study, it was shown that propidium monoazide (PMA) coupled with qPCR detects infectivity of PEDV. The research team optimized the primers, the working concentration of PMA, and the inactivation method using heat or ultraviolet (UV). The results indicated that an optimal plan of PMA could be extremely useful for evaluating infectious and non-infectious viruses (Liang et al.).

The fourth manuscript was “*In vitro evaluation of sodium butyrate on the growth of three Salmonella serovars derived from pigs at a mild acidic pH value*” (Hollmann et al.) by corresponding authors Jan Berend Lingens, from the research team of the University of Veterinary Medicine Hannover, Germany. The research used different sodium butyrate (SB) concentrations (0, 5, 10, 20, 40, and 80 mM) to determine the growth of three different Salmonella enterica serovars (Hollmann et al.). Salmonellosis, as a zoonotic disease, is considered the second most commonly occurring bacterial gastrointestinal disease in humans worldwide (4). Results from the experiment suggested that increasing SB concentrations can suppress Salmonella growth *in vitro* (Hollmann et al.).

The final manuscript was “*Risk factors influencing swine influenza A virus infection in South Korea: a systematic review and meta-analysis of prevalence and seroprevalence*” (Lee et al.) by corresponding author Gayeon Won, from the research team of the College of Veterinary Medicine, Jeonbuk National University, Iksan, South Korea. Swine influenza (SI), a highly infectious viral disease, has a significant impact on the swine industry and on human health worldwide. Due to receptors in the respiratory epithelial cells of pigs, it plays an important role in SI as a mixing vessel for the influenza virus of human, swine, and avian origin (5). A systematic review and meta-analysis based on eight databases were done to investigate the prevalence and seroprevalence of SI in South Korean domestic pigs and to evaluate important risk factors (Lee et al.). Data indicated that the risk of SI circulation was high in the South Korean swine industry. In addition, it was shown that domestic pigs play a key role in the emergence of new types of epidemic zoonoses as mixing vessels for the influenza virus (Lee et al.).

## Author contributions

C-NL, PT, TO, and PTO co-wrote the manuscript. All authors contributed to the article and approved the submitted version.
